# Relationships between Lip Seal Strength, Tongue Pressure, and Daytime Sleepiness in Japanese Workers: A Cross-Sectional Study

**DOI:** 10.3390/clinpract13040068

**Published:** 2023-07-04

**Authors:** Akira Minoura, Yoshiaki Ihara, Hirotaka Kato, Kouzou Murakami, Yoshio Watanabe, Kojiro Hirano, Yoshinori Ito, Akatsuki Kokaze

**Affiliations:** 1 Department of Hygiene, Public Health and Preventive Medicine, Showa University School of Medicine, 1-5-8 Hatanodai, Shinagawa-ku, Tokyo 142-8555, Japan; 2 Division of Oral Functional Rehabilitation Medicine, Department of Special Needs Dentistry, School of Dentistry, Showa University, 2-1-1 Kitasenzoku, Ohta-ku, Tokyo 145-8515, Japan; 3 Department of Radiology, Division of Radiation Oncology, Showa University School of Medicine, 1-5-8 Hatanodai, Shinagawa-ku, Tokyo 142-8555, Japan; 4 Division of Respiratory Medicine and Allergology, Department of Medicine, Showa University School of Medicine, 1-5-8 Hatanodai, Shinagawa-ku, Tokyo 142-8555, Japan; 5 Department of Otorhinolaryngology Head and Neck Surgery, Showa University School of Medicine, 1-5-8 Hatanodai, Shinagawa-ku, Tokyo 142-8555, Japan

**Keywords:** Japanese, lip seal strength, obstructive sleep apnea, tongue pressure, workers

## Abstract

This cross-sectional study aimed to examine the relationships between lip seal strength, tongue pressure, and daytime sleepiness in Japanese workers. A self-administered questionnaire which comprised the Epworth Sleepiness Scale (ESS) was completed by 496 workers, and excessive daytime sleepiness was defined by an ESS score of 11 or over. Lip seal strength and tongue pressure were measured in all participants, and multiple regression analyses were performed to examine the effects of lip seal strength and tongue pressure on daytime sleepiness. The median ESS score was 5.0 (25th and 75th percentiles: 2.0 and 8.0), and 42 (8.5%) workers had excessive daytime sleepiness. The median lip seal strength was 13.5 N (11.4, 16.3), and the tongue pressure was 41.7 kPa (35.2, 48.3). Workers with higher ESS scores had significantly lower levels of lip seal strength than those without, following adjustments for age and body mass index (BMI) values (β (95% confidence interval): −0.043 [−0.081, −0.004]). However, tongue pressure was not significantly associated with ESS score after adjustments for age and/or BMI. These results suggest that maintaining moderate lip seal strength may help prevent excessive daytime sleepiness in Japanese workers regardless of age or BMI.

## 1. Introduction

Daytime sleepiness represents one of the major occupational health issues that directly affects worker performance [1,2]. Although the age-related depression of the root of the tongue is known to promote obstructive sleep apnea, it is thought that understanding age-related changes through dental checkups and other means could directly lead to the prevention of obstructive sleep apnea as subjective symptoms are rare [3]. The early detection and prevention of obstructive sleep apnea are of particular interest to drivers because daytime sleepiness involves risks that directly affect the lives of the workers themselves and their customers [4]. The population of Japan is rapidly aging, which is expected to lead to increasing numbers of not only older workers but also workers with excessive daytime sleepiness. Lee et al. [1] reported that long working times can increase the risk of obstructive sleep apnea, so increased working times for individual workers due to a decreasing working population may also lead to excessive daytime sleepiness. Maintaining a good working environment may reduce the risk of excessive daytime sleepiness and ultimately improve working performance.

Some studies have suggested associations between oral-related indicators and daytime sleepiness. Smardz et al. [5] reported finding no association between obstructive sleep apnea and sleep bruxism. Nakamura et al. [6] found that lip seal strength may be an indicator for assessing mastication and swallowing status, even in healthy young adults. An Asian study reported that both short and long sleep durations were significantly associated with the development of poor oral health status [7].Pediatric research has also suggested that various oral malformations found in children can manifest as sleep disorders in adults [8]. Therefore, it is of great significance to examine the relationship between excessive daytime sleepiness and lip closure force in workers across a wide age range. Although the lips and tongue are composed of multiple muscles, lip seal strength and tongue pressure play important roles in articulation, swallowing, and maintaining dentition [3,5]. The oral health of workers has an effect on not only the prevention of chronic diseases but also the quality of their work [5]. Moreover, some effects in the oral environment have few subjective symptoms, and periodic measurements may lead to the early prevention of chronic diseases. While Kugimiya et al. reported that lip seal strength is affected by aging and was not normally distributed in adults, lip seal strength and tongue pressure were significantly correlated among adults in Japan [9].

We previously examined the lip seal strength and tongue pressure of Japanese workers by age group and found the possibility that lip seal strength and tongue pressure decrease with age, and changes in eating habits affect lip seal strength and tongue pressure in young workers [10]. Since lip seal strength and tongue pressure are closely related to eating habits, it may be possible to demonstrate the prevention of excessive daytime sleepiness by intervening in eating habits via examining the effects of lip seal strength and tongue pressure on sleepiness.

Given these backgrounds, we hypothesized that lip seal strength and tongue pressure are associated with excessive daytime sleepiness in Japanese workers. Therefore, the present study aimed to examine the relationships between lip seal strength, tongue pressure, and daytime sleepiness among Japanese workers.

## 2. Materials and Methods

### 2.1. Study Participants

This study was conducted from November 2021 to June 2022, and the study participants were 496 workers (454 males and 42 females) who were employed at two Japanese companies belonging to the taxi industry. Before the study began, written informed consent was obtained from all participants. Alcohol consumption, smoking, and overweight/obesity have been reported to be associated with non-restorative sleep among healthy Japanese [9], so we administered a self-administered questionnaire composed of items on age, alcohol consumption (once a week or more, less than once a week), smoking (yes or no), weight (kg), height (cm), and shift work (none, less than half the week, or more than half of the week). Due to the COVID-19 pandemic in Japan, job descriptions are fluid, such as changing from driver to clerk, so job descriptions were not included in the questionnaire. This study was approved by the Medical Ethics Committee of Showa University School of Medicine (approval No. 21-088-A, 8 October 2021).

### 2.2. Outcome Variables

In this study, daytime sleepiness was set as the outcome variable. All participants were asked to answer the validated Epworth Sleepiness Scale (ESS) questionnaire, which is composed of eight items (0–3 points per item) that assess the likelihood of falling asleep during a variety of daily living situations. The Epworth sleepiness scale questionnaire (total score range: 0–24 points) was used to assess daytime sleepiness, with higher scores indicating more daytime sleepiness [11]. Normal daytime sleepiness is defined by an ESS score from 0 to 10, and excessive daytime sleepiness is defined by an ESS score of 11 or over (suspected obstructive sleep apnea).

### 2.3. Exposure Variables

**Figure 1:**
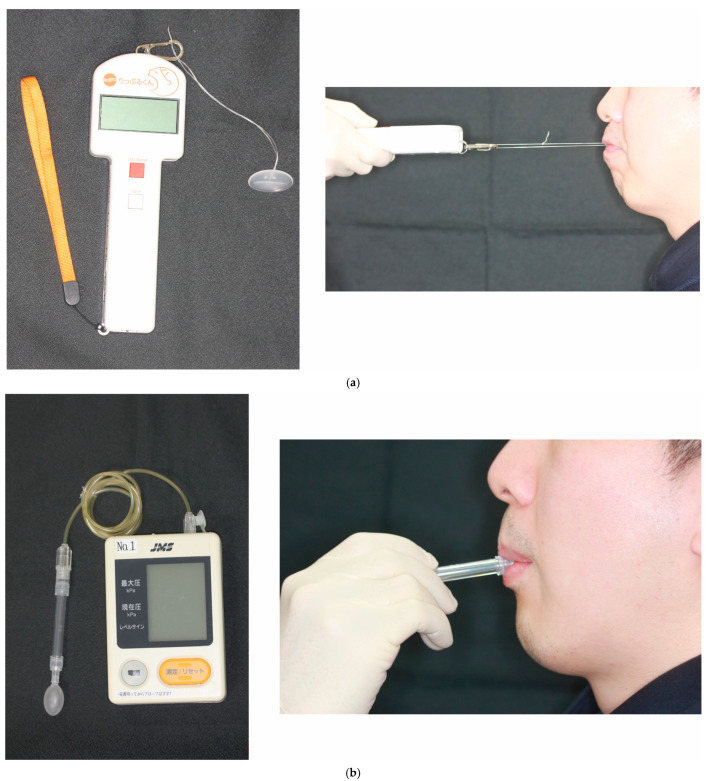
Devices and methods for measuring the lip seal strength and tongue pressure. (**a**) Measurement of lip seal strength. (**b**) Measurement of tongue pressure (the device displays current and maximum pressures).

We assessed lip seal strength and tongue pressure as the exposure variables in all participants. We used a lip seal strength measurement device (Lipple kun, Shofu, Kyoto, Japan) that has been reported to be reliable [12,13,14]. Dental floss (30 cm long) was tied in a ring shape, and a Lipple button (Shofu) was attached to the tip of a Lipple kun [14]. The Lipple kun, which has an oral screen-like shape, was placed in the oral vestibule [14]. This device is shown in Figure 1.

During measurements, the subjects sat in a manner with the head position parallel to the Frankfurt plane and floor. They were also instructed in advance about the following measurement conditions: to resist the traction using only the muscle strength of the lips, not to resist the traction using a sucking force (not to make the intraoral pressure negative), and not to bend the head and body forward while resisting. The examiner stood in front of the subjects and measured their lip seal strength. After confirming that they had placed the Lipple button between the upper and lower anterior tooth regions and the button had been positioned at the center of dentition, all subjects were instructed to close their lips. The Lipple kun was set at a position in the midline of the face and parallel to the floor to irradiate the subnasale with the indicator light. Then, the button was pulled slowly in the horizontal direction while maintaining the position of the Lipple kun. The button was pulled until it was out of the lips, and the maximum value during this period was recorded using the Lipple kun. The subjects practiced this procedure once prior to measurement. Measurements were performed after confirming that the subject had sufficiently understood the measurement method. Measurements were repeated three times while ensuring that a break was taken, and the mean value was defined as the lip seal strength (N).

We used a tongue pressure measuring device (TPM-01; JMS Co. Ltd., Hiroshima, Japan) to evaluate tongue pressure. An intraoral balloon-shaped probe was placed on the center of the tongue and behind the upper incisors, on the hard palate. During all measurements, the participants were instructed to close their lips, press their tongues against the hard palate, and push the probe with maximum tongue force. Next, the maximum air pressure of the probe was recorded using the device. At the same time, the device displayed the current and maximum pressures. This device is shown in Figure 1. The tongue pressure was recorded three times, and the mean value was adopted as the participant’s tongue pressure. Each measurement was made after at least 1 min of rest from the last measurement [15].

### 2.4. Statistical Analysis

We performed multiple regression analyses to examine the effects of lip seal strength and tongue pressure on excessive daytime sleepiness. Before performing the analyses, we calculated correlation coefficients (Pearson’s r) to check for multicollinearity among the ESS score, lip seal strength, tongue pressure, age, and BMI. The results were expressed as β with 95% confidence intervals (CIs). A Shapiro–Wilk test was performed for continuous variables to confirm normal contribution. For continuous variables that were not normally distributed, median values (25th and 75th percentiles) are shown instead of mean values. A multiple regression analysis was performed for continuous variables. We defined *p* values < 0.05 as statistically significant. This study followed the Strengthening the Reporting of Observational studies in Epidemiology (STROBE) guideline for cross-sectional studies. All statistical analyses were conducted using JMP 16.2 (SAS Institute, Inc., Cary, NC, USA).

## 3. Results

A total of 496 workers were included in the analysis. The characteristics of the participants are shown in Table 1. The results of the self-administered questionnaire indicated that 60 (12.1%) workers had daytime sleepiness. There was no significant difference between shift work and no shift work in ESS scores and daytime sleepiness among the study participants. Among all workers, the median lip seal strength was 13.5 N (25th and 75th percentiles: 11.4 and 16.3), and the median tongue pressure was 41.7 kPa (35.2, 48.3). Although 57.7% of the workers consumed alcohol, there was no significant difference between the presence or absence of daytime sleepiness (*p* < 0.911). Also, although 35.5% of the workers smoked, there was no significant difference between the presence or absence of daytime sleepiness (*p* < 0.480). Among workers without daytime sleepiness, the median lip seal strength was 13.5 N (11.5, 16.3), and the median tongue pressure was 41.8 kPa (35.9, 48.5). Among workers with daytime sleepiness, the median lip seal strength was 12.7 N (10.8, 15.4), and the median tongue pressure was 38.5 kPa (32.5, 45.8). Compared to workers without daytime sleepiness, workers with daytime sleepiness had significantly lower levels of lip seal strength (*p* = 0.028) and tongue pressure (*p* = 0.003), respectively. On the other hand, there was no significant difference in BMI, alcohol consumption, and smoking between workers with daytime sleepiness and workers without daytime sleepiness. As results of the Shapiro–Wilk tests, all continuous variables were confirmed to not be normally distributed.

Table 2 shows the correlation coefficients between the Epworth sleepiness scale scores and the covariates. The correlation coefficients among the Epworth sleepiness scale scores ranged from 0.052 to 0.393, and the ESS scores significantly correlated with lip seal strength, tongue pressure, and age, respectively. Lip seal strength was correlated with tongue pressure. As all covariates were not strongly correlated with any of the other covariates, we did not exclude any covariates from the analyses to avoid multicollinearity.

Table 3 summarizes the association between ESS score and lip seal strength according to the multiple regression analysis among all participants. In this analysis, we used four models to examine the effects of age and BMI on lip seal strength and daytime sleepiness. Model 1 was a multiple regression analysis of daytime sleepiness and oral health indicators which excluded age and BMI. Model 2 was a multiple regression analysis of daytime sleepiness and oral health indicators which excluded age. Model 3 was a multiple regression analysis of daytime sleepiness and oral health indicators which excluded BMI. Model 4 was a multiple regression analysis of daytime sleepiness and oral health indicators. Lip seal strength was found to be positively associated with ESS scores after adjusting for sex, alcohol consumption, and smoking (β (95%CI): −0.113 (−0.223, −0.003)). However, this association was not significant after adjusting for age and/or BMI. Table 4 summarizes the association between ESS scores and tongue pressure according to the multiple regression analysis among workers. In this analysis, we used four models to examine the effects of age and BMI on tongue pressure and daytime sleepiness. Model 1 was a multiple regression analysis of daytime sleepiness and oral health indicators which excluded age and BMI. Model 2 was a multiple regression analysis of daytime sleepiness and oral health indicators which excluded age. Model 3 was a multiple regression analysis of daytime sleepiness and oral health indicators which excluded BMI. Model 4 was a multiple regression analysis of daytime sleepiness and oral health indicators. After adjusting for sex, alcohol consumption, and smoking, tongue pressure was found to be positively associated with ESS scores (−0.043 (−0.081, −0.004)). This association remained significant after adjusting for age and/or BMI.

Table 5 summarizes the associations between lip seal strength, tongue pressure, and excessive daytime sleepiness. We performed logistic regression analyses to examine independently the effects of lip seal strength and tongue pressure. Analyses of lip seal strength in tertiles (low, medium, and high) showed that workers with high or low lip seal strength were not significantly associated with excessive daytime sleepiness. Analyses of tongue pressure in tertiles (low, medium, and high) showed that workers with high or low tongue pressure were not significantly associated with excessive daytime sleepiness.

## 4. Discussion

After adjusting for age, sex, alcohol consumption, smoking, and BMI, the results of the present study suggest that lower tongue pressure may lead to daytime sleepiness but may not associate with excessive daytime sleepiness among Japanese workers. On the other hand, lip seal strength was not significantly associated with daytime sleepiness after adjusting for age and/or BMI. These results suggest that lip seal strength may be related to workers’ daytime sleepiness, but there was no association with excessive daytime sleepiness.

As previous studies have suggested that daytime sleepiness related to obstructive sleep apnea may be prevented by improving the working environment [16], the results of this study further suggest that both improving oral health and examining age-related changes in lip seal strength may relate to daytime sleepiness among workers. Although the percentage of workers with daytime sleepiness in this study was 12.1%, the results were consistent with previous studies and were considered adequate for analysis [17,18]. With the proportion of the older population rapidly increasing in Japan, the government is promoting the employment of the aged [19]. As the depression of the tongue root due to aging is known to promote the development of obstructive sleep apnea, examining age-related changes in lip seal strength and tongue pressure may help prevent sleep disorders among workers [3]. In particular, for drivers, the early detection and prevention of obstructive sleep apnea are key aspects of occupational health because disordered sleep involves risks that can directly affect the lives of the workers themselves and their customers. Moreover, metabolic syndrome, a risk factor for obstructive sleep apnea, is reported to have a prevalence of about 20%; however, to our knowledge, there are no reports on sex differences, severity, and its effects in work systems, especially for occupational drivers and shift workers in small and medium-sized companies [13]. Therefore, it is important to measure lip seal strength to help predict obstructive sleep apnea and excessive daytime sleepiness, especially in occupations that perform life-threatening tasks, such as drivers.

Many epidemiologic studies have suggested that daytime sleepiness related to obstructive sleep apnea is a risk factor for insulin resistance [20]. Excessive daytime sleepiness has also been associated with insulin resistance in case–control studies of young, healthy, non-obese men; thus, daytime sleepiness may contribute to insulin resistance independent of age and obesity [21]. Although obstructive sleep apnea and type 2 diabetes share the common risk factors of obesity and aging, oral health may also play a role. Future studies should explore causal inferences that include eating habits, daytime sleepiness, type 2 diabetes, and indicators of the oral environment, including lip seal strength and tongue pressure. Lip seal strength, tongue pressure, and eating habits can be improved with interventions and thus may lead to the prevention of both obstructive sleep apnea and type 2 diabetes.

Patients with obstructive sleep apnea have been reported to have smaller anatomic upper airway diameters compared with healthy individuals [22,23]. Smaller and heritable craniofacial dimensions have been reported to predispose nonobese individuals to daytime sleepiness related to obstructive sleep apnea [24]. Therefore, the possibility that weak lip seal strength may lead to daytime sleepiness is fully considered and consistent with the results of the present study.

On the other hand, measuring the oral environment over the long term is considered an important concept for the prevention of excessive daytime sleepiness. Sasakawa et al. [25] reported that lip seal strength may vary among healthy children depending on what they eat during meals. It is possible that lip seal strength in adulthood may be affected by eating behavior in childhood and should therefore be examined in future studies. Future studies should also examine how lifestyle habits in childhood and other factors may affect daytime sleepiness in adulthood. As the COVID-19 pandemic has adversely affected the treatment and diagnosis of obstructive sleep apnea, Miller et al. [26] conducted a systematic review and suggested the need to manage obstructive sleep apnea using a variety of methods. The association between obstructive sleep apnea and lip closure strength found in the present study may lead to the improvement of such a management system. Another recent study reported the validity of ESS scores for assessing sleepiness in children [27]. It may therefore be possible to prevent and improve sleep disorders more effectively by focusing on ESS scores and lip seal strength from childhood.

Sleep disorder can lead to serious health problems associated with excessive daytime sleepiness and is known to be associated with morbidities such as hypertension and cerebral vascular disease [28]. Therefore, in Japan, where the working population is expected to decrease because of the increasingly low birth rate and aging population, it is urgent to secure the labor force through the prevention of or early intervention for obstructive sleep apnea. The results of this study may help establish new methods to aid in the prevention of or early intervention for daytime sleepiness. In this study, we decided to determine and analyze the epidemiologic profiles of workers with obstructive sleep apnea from oral health checkups because a lack of subjective symptoms led to the possibility that many obstructive sleep apnea patients were undiagnosed.

This study had several limitations. First, because it was designed as a cross-sectional study, we were not able to examine causal relationships between sleep disorders (obstructive sleep apnea or/and daytime sleepiness) and oral health (lip seal strength or/and tongue pressure). It is necessary to study the mechanisms of how the tongue and lips affect sleep among workers from different perspectives. Second, due to the small number of female workers with excessive daytime sleepiness, sex differences in oral health and sleep disorders could not be investigated. Some occupational health studies have indicated the presence of sex differences in the magnitude and directional specificity of the lip seal strength produced during pursing, like lip closing movements, in healthy young adults [29,30]. While these studies have reported sex differences in lip seal strength, a future survey should be conducted with more participants to investigate sex differences in sleep disorders and oral health. Third, the impact of the COVID-19 pandemic on workers’ sleep could not be assessed in this study. It is possible that changes in workers’ lifestyles (especially in terms of BMI and dietary habits such as exercise, nutrition from diet, and overeating) due to COVID-19 prevention measures may have had effects on sleep disorders and the measurement of oral functions [31,32]. While previous studies have shown that obstructive sleep apnea patients with medical diagnoses have higher ESS scores than those without obstructive sleep apnea, our results suggest that there was no significant difference in ESS scores between suspected obstructive sleep apnea workers and non-OSA workers. With treatment, patients with obstructive sleep apnea may show improved sleep quality that is comparable to or better than those without obstructive sleep apnea, which must be investigated in future longitudinal studies. In any case, it is too difficult to diagnose obstructive sleep apnea in a large number of people during the COVID-19 pandemic, and in this study, sleep disorder was assessed through the use of a self-administered questionnaire, as in previous studies during a pandemic [18]. Therefore, lip seal strength and tongue pressure should be further investigated after the COVID-19 pandemic. Future studies should investigate the causal relationships between lip seal strength, tongue pressure, and sleep disorders and the effects of the COVID-19 pandemic by conducting longitudinal studies among Japanese workers.

## 5. Conclusions

The results of the present study suggest that maintaining moderate lip seal strength may related to prevention of daytime sleepiness but is not related to excessive daytime sleepiness among Japanese workers. The findings also suggest that measuring lip seal strength may enable the early detection and prevention of daytime sleepiness that affects performance among workers.

## Figures and Tables

**Table 1:** Characteristics of study participants.

	Total	Daytime Sleepiness−	Daytime Sleepiness+ ^1^	*p*-Value ^2^
	(N = 496)	(N = 436 [87.9%])	(N = 60 [12.1%])	
Lip seal strength [N]	13.5 (11.4, 16.3)	13.5 (11.5, 16.3)	12.7 (10.8, 15.4)	0.028
Tongue pressure [kPa]	41.7 (35.2, 48.3)	41.8 (35.9, 48.5)	38.5 (32.5, 45.8)	0.003
ESS score (range: 0–24)	5.0 (2.0, 8.0)	4.0 (2.0, 7.0)	13.0 (12.0, 15.0)	<0.001
Age	47.0 (30.0, 55.0)	47.0 (32.0, 55.0)	44.0 (26.0, 54.0)	0.143
Male	454 (91.5)	403 (92.4)	51 (85.0)	0.053
BMI	24.1 (21.3, 27.0)	24.2 (21.5, 27.0)	23.6 (20.7, 27.1)	0.076
Overweight or obese ^3^	202 (40.7)	182 (41.7)	20 (33.3)	0.214
Alcohol consumption	286 (57.7)	251 (57.6)	35 (58.3)	0.911
(once a week or more)				
Smoking	176 (35.5)	158 (36.2)	18 (30.0)	0.480
(yes)				

Except where indicated, n (%), values are medians (25th and 75th percentiles). ESS: Epworth Sleepness Scale; BMI: body mass index. ^1^ Defined by an ESS score of 11 or over. ^2^ Wilcoxon rank-sum test or Fisher’s exact test. ^3^ Defined by a BMI of 25 or over.

**Table 2:** Correlation coefficients between ESS and covariates.

				Pearson’s r					
	a	b		c		d		e	
a. ESS score		−0.131	*	−0.121	*	−0.148	*	−0.052	
b. Lip seal strength				0.393	*	0.295	*	0.317	*
c. Tongue pressure						0.052		0.285	*
d. Age								0.214	*
e. BMI									

ESS: Epworth Sleepiness Scale; BMI: body mass index. * Statistically significant (*p*-value < 0.05).

**Table 3:** Association between ESS score and lip seal strength.

	Model 1	Model 2	Model 3	Model 4
	β (95%CI)	β (95%CI)	β (95%CI)	β (95%CI)
Lip seal strength	−0.105 (−0.208, −0.002) *	−0.102 (−0.210, 0.006)	−0.078 (−0.184, 0.028)	−0.079 (−0.189, 0.031)
Age			−0.029 (−0.056, −0.001) *	−0.029 (−0.057, −0.001) *
BMI		0.008 (−0.098, 0.081)		0.004 (−0.086, 0.094)
Male	−0.973 (−1.645, −0.301) *	−0.970 (−1.643, −0.297) *	−0.861 (−1.539, −0.182) *	−0.861 (−1.541, −0.182) *
Alcohol use	0.148 (−0.220, 0.515)	0.150 (−0.218, 0.519)	0.125 (−0.242, 0.492)	0.124 (−0.245, 0.492)
Smoking	0.300 (−0.081, 0.681)	0.300 (−0.081, 0.681)	0.244 (−0.160, 0.649)	0.293 (−0.087, 0.673)

BMI: body mass index; β: Standardized partial regression coefficient; Model 1: Multiple regression analysis of ESS score and oral health indicators which excluded age and BMI; Model 2: Multiple regression analysis of ESS score and oral health indicators which excluded age; Model 3: Multiple regression analysis of ESS score and oral health indicators which excluded BMI; Model 4: Multiple regression analysis of ESS score and oral health indicators. * Statistically significant (*p*-value < 0.05).

**Table 4:** Association between ESS score and tongue pressure.

	Model 1	Model 2	Model 3	Model 4
	β (95%CI)	β (95%CI)	β (95%CI)	β (95%CI)
Tongue pressure	−0.042 (−0.079, −0.005) *	−0.041 (−0.080, −0.002) *	−0.042 (−0.083, −0.002) *	−0.043 (−0.081, −0.004) *
Age			−0.033 (−0.061, −0.004) *	−0.034 (−0.061, −0.006) *
BMI		−0.009 (−0.097, 0.080)		0.014 (−0.076, 0.104)
Male	−1.023 (−1.684, −0.361) *	−1.018 (−1.682, −0.354) *	−0.842 (−1.533, −0.150) *	−0.853 (−1.527, −0.179) *
Alcohol use	0.175 (−0.192, 0.542)	0.177 (−0.191, 0.545)	0.131 (−0.259, 0.520)	0.140 (−0.227, 0.507)
Smoking	0.313 (−0.067, 0.693)	0.313 (−0.068, 0.693)	0.249 (−0.154, 0.652)	0.296 (−0.082, 0.675)

BMI: body mass index; β: standardized partial regression coefficient. * Statistically significant (*p*-value < 0.05).

**Table 5:** Association between lip seal strength, tongue pressure, and excessive daytime sleepiness.

		Model 1	Model 2	Model 3
		OR (95%CI)	OR (95%CI)	OR (95%CI)
Lip seal strength	Low	1.21 (0.63–2.32)		1.12 (0.58–2.17)
	Medium	Ref.		Ref.
	High	0.88 (0.43–1.79)		0.98 (0.47–2.02)
Tongue pressure	Low		1.50 (0.78–2.86)	1.47 (0.76–2.83)
	Medium		Ref.	Ref.
	High		0.82 (0.39–1.69)	0.83 (0.40–1.74)

All models were adjusted for age, gender, overweight or obese, alcohol use, and smoking. Lip seal strength and tongue pressure were categorized via tertiles (low, medium, and high). OR: odds ratio; CI: confidence interval.

## Data Availability

In this study, the data are not available in a public repository but are available from the corresponding author (Akira Minoura) upon reasonable request.
